# Effect of the Decrease in Luminance Noise Range on Color Discrimination of Dichromats and Trichromats

**DOI:** 10.3389/fnbeh.2018.00292

**Published:** 2018-11-23

**Authors:** Terezinha Medeiros Gonçalves de Loureiro, Ketan Brodeur, Genevieve Schade, Felipe André Costa Brito, Railson Cruz Salomao, Leticia Miquilini, Daniela Maria Oliveira Bonci, Luiz Claudio Portnoi Baran, Einat Hauzman, Paulo Roney Kilpp Goulart, Maria Izabel Tentes Cortes, Dora Fix Ventura, Malinda E. C. Fitzgerald, Givago Silva Souza

**Affiliations:** ^1^Instituto de Ciências Biológicas, Universidade Federal do Pará, Belém, Brazil; ^2^University of Michigan, Ann Arbor, MI, United States; ^3^Southern College of Optometry, Memphis, TN, United States; ^4^Núcleo de Medicina Tropical, Universidade Federal do Pará, Belém, Brazil; ^5^Instituto de Psicologia, Universidade de São Paulo, São Paulo, Brazil; ^6^Núcleo de Teoria e Pesquisa do Comportamento, Universidade Federal do Pará, Belém, Brazil; ^7^Centro de Ciências da Saúde, Universidade Federal do Amapá, Macapá, Brazil; ^8^Biology, Didactic Faculty and Anatomy and Neurobiology, Christian Brothers University, Memphis, TN, United States; ^9^University of Tennessee Health Science Center, Memphis, TN, United States

**Keywords:** color vision, color discrimination, luminance noise, trichromats, dichromats

## Abstract

Color vision assessment can be done using pseudoisochromatic stimuli, which has a luminance noise to eliminate brightness differences between the target and background of the stimulus. It is not clear the influence of the luminance noise on color discrimination. We investigated the effect of change in the luminance noise limits on color discrimination. Eighteen trichromats and ten congenital dichromats (eight protans, two deutans) had their color vision evaluated by the Cambridge Colour Test, and were genetically tested for diagnostic confirmation. The stimuli were composed of a mosaic of circles in a 5° circular field. A subset of the circles differed in chromaticity from the remaining field, forming a letter C. Color discrimination was estimated in stimulus conditions differing in luminance noise range: (i) 6–20 cd/m^2^; (ii) 8–18 cd/m^2^; (iii) 10–16 cd/m^2^; and (iv) 12–14 cd/m^2^. Six equidistant luminance values were used within the luminance noise limits with the mean stimulus luminance maintained constant under all conditions. A four-alternative, forced-choice method was applied to feed a staircase procedure to estimate color discrimination thresholds along eight chromatic axes. An ellipse model was adjusted to the eight color discrimination thresholds. The parameters of performance were threshold vector lengths and the ellipse area. Results were compared using the Kruskal-Wallis test with a significance level of 5%. The linear function model was applied to analyze the dependence of the discrimination parameters on the noise luminance limits. The first derivative of linear function was used as an indicator of the rate of change in color discrimination as a function of luminance noise changes. The rate of change of the ellipse area as a function of the luminance range in dichromats was higher than in trichromats (*p* < 0.05). Significant difference was also found for individual thresholds in half of the axes we tested. Luminance noise had a greater effect on color discrimination ability of dichromats than the trichromats, especially when the chromaticities were close to their protan and deutan color confusion lines.

## Introduction

Several psychophysical protocols for assessment of color discrimination have been developed using pseudoisochromatic design, such as the Ishihara Test, the AO HRR pseudoisochromatic plates and the Cambridge Colour Test (Regan et al., 1994). This approach allows elimination contour and luminance cues from the presentation of chromatic stimuli. It is therefore important for the unbiased evaluation of color vision necessary in research and in diagnosis of congenital or acquired color vision losses (Regan et al., 1994; Moura et al., 2008; Paramei, 2012; Paramei and Oakley, 2014; Shinomori et al., 2016).

Pseudoisochromatic stimulus, usually, are composed by a mosaic of circles of different sizes (spatial noise) and luminance (luminance noise). Changes in luminance noise impact color discrimination thresholds (Souza et al., 2014; Cormenzana Méndez et al., 2016; Linhares et al., 2016) and the decrease in number of noise luminance values and the increase in contrast between luminance noise and the mean luminance impaired the color discrimination thresholds (Souza et al., 2014; Cormenzana Méndez et al., 2016). Linhares et al. (2016) investigated the influence of the dynamic luminance contrast noise (flickering at 10 Hz) on the color discrimination thresholds of normal trichromats and anomalous trichromats. They observed that the presence of the dynamic noise had no effect on the color discrimination thresholds of normal trichromats but improved the color discrimination thresholds of the anomalous trichromats. These findings indicate the need to understand how several parameters of the pseudoisochromatic stimulus could influence color vision and to guide future protocol standardization that would permit comparisons between laboratories.

The present investigation aims to extend the previous studies on the influence of changes in luminance noise parameters on the color discrimination thresholds. We changed the luminance noise range of the stimulus, without changing the mean luminance or luminance noise level, to estimate the color discrimination thresholds of normal trichromats and congenital dichromats. We observed that luminance noise had a greater effect on the color discrimination ability of the dichromats than the trichromats.

## Material and Methods

### Subjects

The participants were 18 trichromats (13 female, 5 male, 25.55 ± 1.5 years-old) and 10 congenital dichromats (seven male and one female protans, 24.75 ± 4.5 years-old; two male deutans, 24.5 ± 2.1 years-old). All individuals had normal visual acuity or corrected to 20/20 with no history of visual or neurological diseases. The authors evaluated the red-green color vision genotype of those participants that has color discrimination performance, suggestive of color deficiency in the Cambridge Colour Test and Ishihara assessment. All participants gave written informed consent to participate in the experiments, and the Ethical Committee for Research in Humans approved the experimental procedures (Center of Tropical Medicine, Federal University of Pará, Brazil, report #570.434).

### Apparatus

We used the Cambridge Colour Test (Cambridge Research System, CRS, Rochester, United Kingdom; CCT) to estimate the color discrimination thresholds of participants. The test ran in the ViSaGe system (CRS) with gamma-correct driven CRT display (spatial resolution: 1600 × 1200 pixels, temporal resolution: 75 Hz, color depth: 14 bits per gun; Diamond Pro 2070SB model, Mitsubishi, Australia). The gamma correction was done using vsgDesktop software, version 2.10 (CRS) and ColorCal chromameter (CRS).

**Figure 1:**
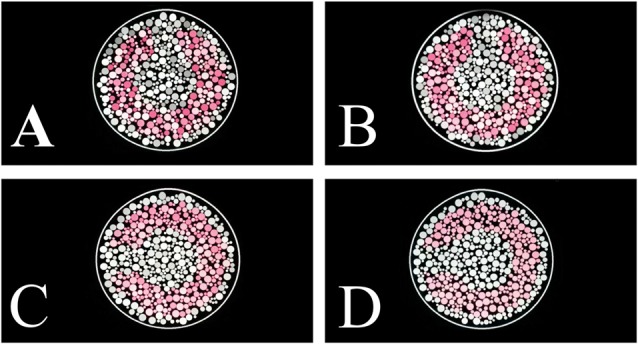
Pseudoisochromatic stimulus examples with different luminance noise range conditions. **(A)** Luminance noise ranged between 6 cd/m^2^ and 20 cd/m^2^ (amplitude of 14 cd/m^2^). **(B)** Luminance noise ranged between 8 cd/m^2^ and 18 cd/m^2^ (amplitude of 10 cd/m^2^). **(C)** Luminance noise ranged between 10 cd/m^2^ and 16 cd/m^2^ (amplitude of 4 cd/m^2^). **(D)** Luminance noise ranged between 12 cd/m^2^ and 14 cd/m^2^ (amplitude of 2 cd/m^2^).

A mosaic of circles in a 5° circular patch composed the stimulus (Figure 1). The circles had different sizes and luminance. We used four different stimulus conditions differing in luminance noise range: (i) luminance noise between 6 cd/m^2^ and 20 cd/m^2^ (range of 14 cd/m^2^); (ii) luminance noise between 8 cd/m^2^ and 18 cd/m^2^ (range of 10 cd/m^2^); (iii) luminance noise between 10 cd/m^2^ and 16 cd/m^2^ (range of 6 cd/m^2^); and (iv) luminance noise between 12 cd/m^2^ and 14 cd/m^2^ (range of 2 cd/m^2^). We kept the same mean luminance at all stimulus conditions (13 cd/m^2^). Figure 1 shows examples of stimuli for each luminance noise range. Six luminance values composed the luminance noise.

**Figure 2:**
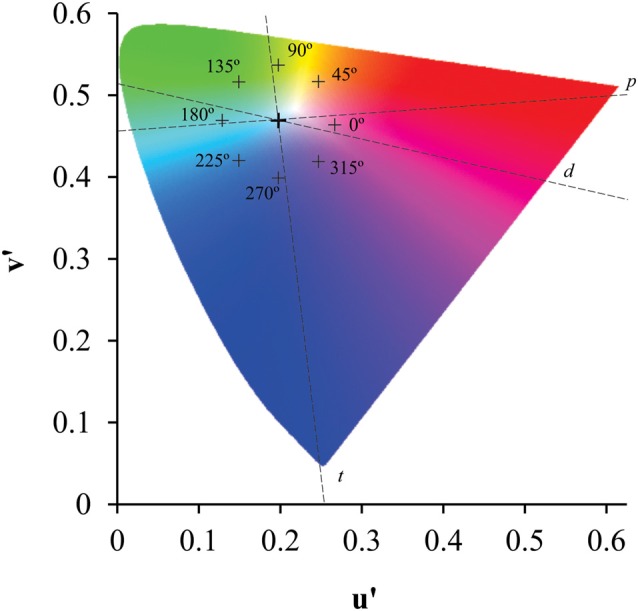
Location of the target chromaticities in the CIE 1976 color diagram. Eight chromatic axes, equally spaced in 45° related to a horizontal axis that cross the reference chromaticity coordinate, had the length controlled by a staircase protocol to estimate the color discrimination threshold related to the background chromaticity. Crosses represent the greater chromatic vector in each axis. Dotted lines represent the protan (*p*), deutan (*d*) and tritan (*t*) confusion lines.

A subset of circles (target) in the shape of a Landot-C (outer diameter: 4.4°; inner diameter: 3.3°; C gap: 1°) differed in chromaticity from the background circles. The chromaticity of the field (CIE1976 color space, u’ = 0.1977 e v’ = 0.4689) was compared to the target chromaticity that could be presented at eight different chromatic axes as shown in the Figure 2. The observer’s task was to indicate the orientation of the C-gap (right, left, top and down). The threshold estimation was conducted using a four-alternative, forced-choice method and a staircase controlled the distance between the field and target chromaticities in the CIE1976 color space. The rule of the staircase case was 1 hit:1 error, and after 10 reversals the procedure was terminated. The average of the last six reversals was used to estimate the color discrimination thresholds.

### Data Analysis

We fitted an ellipse model using the Khachiyan Ellipsoid Method (Khachiyan, 1979) with programmed routines in MATLAB language environment (Mathworks, Natick, MA, USA). We considered the ellipse area and the color discrimination threshold vector lengths as indicators of the psychophysical performance of the chromatic discrimination task. Thresholds were expressed relative to the largest threshold value obtained among the four-luminance noise conditions.

We assessed the relative threshold distribution using D’Agostino-Pearson omnibus test. Kruskal-Wallis test was used to compare the results obtained by the different stimulus conditions for dichromats and trichromats. This was followed by the Dunn’s multiple comparisons test adjusting the significance level for the multiple comparisons. We fitted a straight-line function to the thresholds as a function of the luminance noise amplitude. The slope of the best-fit straight-line function quantified the influence of luminance noise range upon the chromatic thresholds. We evaluated the linear correlation coefficient, coefficient of determination, and the *p*-value of the linear regressions. Significance was considered if *p* < 0.05).

### Color Vision Phenotype and Genotype

The color vision phenotype was defined by the combined evaluation of the CCT in the luminance range of the luminance noise between 8 cd/m^2^ and 18 cd/m^2^ and the genetic evaluation verification. The authors only evaluated the red-green color vision genotype of those participants that had color discrimination performance suggestive of color deficiency in the CCT and Ishihara Test. A total of 10 subjects were genetically evaluated. We extracted DNA samples from buccal swabs using the GentraPuregene Buccal Cell Kit (Gentra Systems, Inc., Minneapolis, MN, USA). The presence of L-opsin and M-opsin genes was investigated in the X chromosome array (Neitz and Neitz, 1995). Blood samples from trichromat, protanope and deuteranope subjects were used as either negative or positive controls.

**Table 1:** Results of the linear regression between chromatic thresholds and luminance noise amplitude.

	Slope (95%CI)	*r*	*r*^2^	*p*
**Trichromats**
**Ellipse area**	**0.021 (0.013–0.028)**	**0.54**	**0.29**	**0.001**
Vector 0° length	0.004 (−0.004–0.012)	0.11	0.013	0.344
**Vector 45° length**	**0.013 (0.003–0.021)**	**0.31**	**0.096**	**0.008**
**Vector 90° length**	**0.013 (0.005–0.021)**	**0.36**	**0.1302**	**0.002**
Vector 135° length	0.006 (−0.001–0.014)	0.19	0.036	0111
Vector 180° length	0.0074 (−0.005–0.015)	0.22	0.047	0.066
**Vector 225° length**	**0.012 (0.005–0.019)**	**0.36**	**0.127**	**0.002**
Vector 270° length	0.006 (−0.002–0.014)	0.17	0.029	0.1476
**Vector 315° length**	**0.012 (0.004–0.019)**	**0.35**	**0.125**	**0.002**
**Dichromats**
**Ellipse area**	**0.036 (0.021–0.051)**	**0.61**	**0.378**	**0.0001**
**Vector 0° length**	**0.030 (0.014–0.046)**	**0.52**	**0.274**	**0.0005**
**Vector 45° length**	**0.024 (0.004–0.038)**	**0.45**	**0.199**	**0.002**
**Vector 90° length**	**0.022 (0.008–0.036)**	**0.50**	**0.254**	**0.0033**
**Vector 135° length**	**0.020 (0.004–0.035)**	**0.37**	**0.140**	**0.017**
Vector 180° length	0.016 (−0.006 to 0.038)	0.22	0.052	0.158
**Vector 225° length**	**0.019 (0.005–0.032)**	**0.41**	**0.172**	**0.008**
Vector 270° length	0.004 (−0.016–0.023)	0.06	0.004	0.690
**Vector 315° length**	**0.018 (0.003–0.032)**	**0.37**	**0.140**	**0.017**

*95% CI: 95% confidence interval; r = correlation coefficient; r^2^ = coefficient of determination; *p* = *p*-value. Significant values are in bold*.

## Results

### Color Discrimination Thresholds as a Function of the Luminance Noise Amplitude

**Figure 3:**
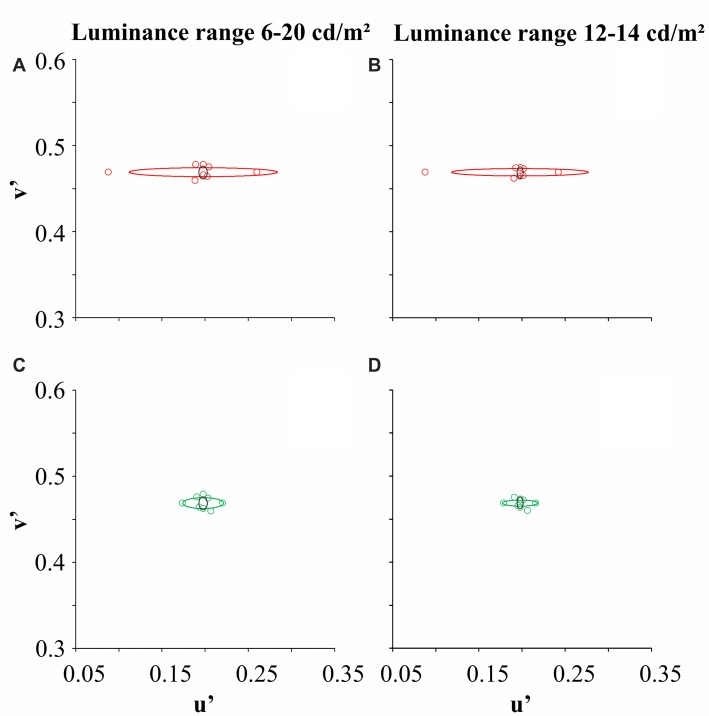
Color discrimination ellipses for one trichromat (black ellipse), one protan subject (red ellipse) and one deutan subject (green circle). Panels **(A,C)** represent color discrimination in the luminance noise amplitude of 14 cd/m^2^, while **(B,D)** represent color discrimination in the luminance noise amplitude of 2 cd/m^2^. Circles represent the threshold coordinates for the protanope (red circles) and deuteranope (green circles).

The genetic analysis of the six dichromat subjects confirmed the CCT results. Figure 3 illustrates the color discrimination ellipses of a trichromat and dichromats (one protan and one deutan subject) for two luminance noise ranges (6–20 cd/m^2^ and 12–14 cd/m^2^).

**Figure 4:**
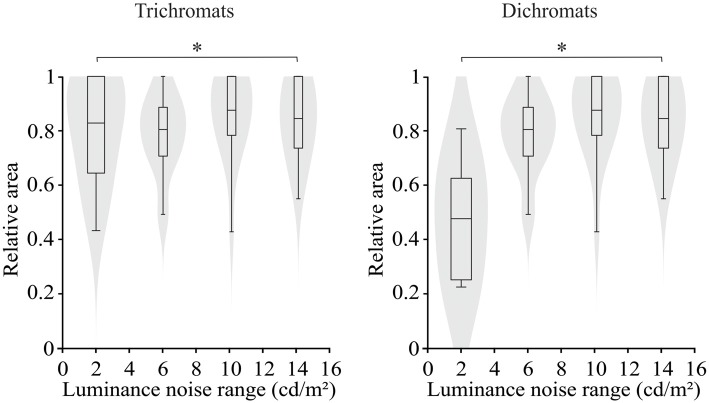
Violin plots of relative ellipse area estimated from trichromats and congenital dichromats as a function of the luminance noise amplitude. Low luminance noise amplitude improved the color discrimination compared to high luminance noise amplitude. Shaded gray areas are the kernel density plot that represents 95% of the data distribution, and the box plot comprises horizontal lines that represent third quartile (upper line), median (middle line), first quartile (lower line) and whiskers represents the maximum and minimum values. *p < 0.05.

For both trichromats and dichromats, there was statistically significant difference between the areas of the ellipses estimated by the luminance noise stimulus condition (*H*_(7)_ = 37.96, *p* < 0.001). The ellipse area at the luminance noise range of 2 cd/m^2^ was smaller than in the luminance noise range of 14 cd/m^2^ (Figure 4, for trichromats: adjusted-*p* value = 0.04; for dichromats: adjusted-*p* value = 0.007).

**Figure 5:**
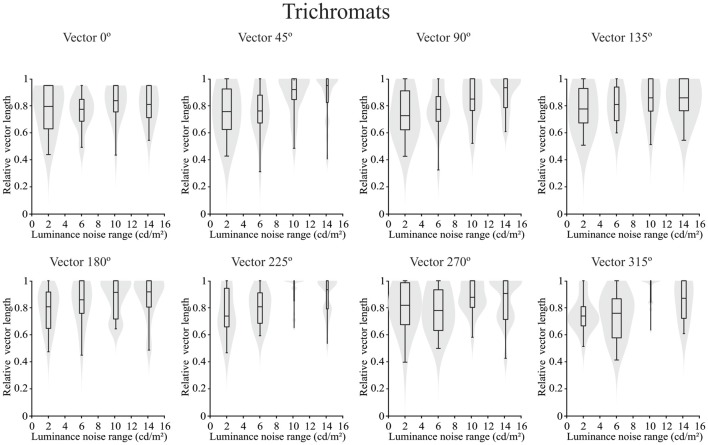
Violin plots of chromatic thresholds estimated from trichromats as a function of the luminance noise amplitude at each chromatic vector. No difference was found in the multiple comparisons from trichromats data. Shaded gray areas are the kernel density plot that represents 95% of the data distribution, and the box plot comprises horizontal lines that represent third quartile (upper line), median (middle line), first quartile (lower line) and whiskers represents the maximum and minimum values.

**Figure 6:**
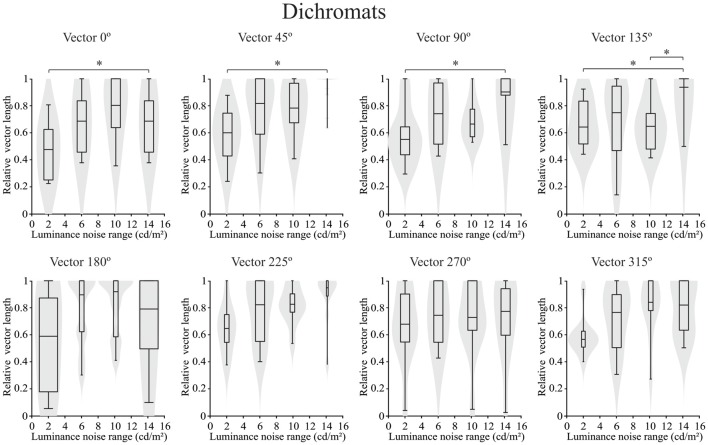
Violin plots of chromatic thresholds estimated from congenital dichromats as a function of the luminance noise amplitude at each chromatic vector. There was difference between luminance noise amplitude conditions of 14 cd/m^2^ and 2 cd/m^2^ at the chromatic axes 0°, 45°, 90° and 135° from dichromats data. Here again the low luminance noise amplitude improved the color discrimination compared to high luminance noise amplitude. Shaded gray areas are the kernel density plot that represents 95% of the data distribution, and the box plot comprises horizontal lines that represent third quartile (upper line), median (middle line), first quartile (lower line) and whiskers represents the maximum and minimum values. *p < 0.05.

Concerning the chromatic vector lengths, it was found that there was no statistically significant difference in the trichromats (*p* > 0.05) for chromatic thresholds estimated by the luminance noise stimulus at any of the different chromatic axes (Figure 5). Conversely a statistically significant differences (*p* < 0.05) was observed in the dichromats for the chromatic thresholds estimated by the luminance noise condition at four chromatic axes (0°, 45°, 90° and 135°) (Figure 6). For dichromats, at the chromatic axis 0°, the threshold vector length was smaller at the luminance noise amplitude of 2 cd/m^2^ than at luminance noise amplitude at 14 cd/m^2^ (Dunn’s multiple comparison test, adjusted-*p* value < 0.05). Additionally, we observed at the chromatic axis 135°, the threshold vector length at the luminance noise range of 10 cd/m^2^ was also smaller than at the luminance noise range of 14 cd/m^2^ (Dunn’s multiple comparison test, adjusted-*p* value < 0.05).

### Linear Regression Analysis of the Chromatic Thresholds as a Function of Luminance Noise Amplitudes

**Figure 7:**
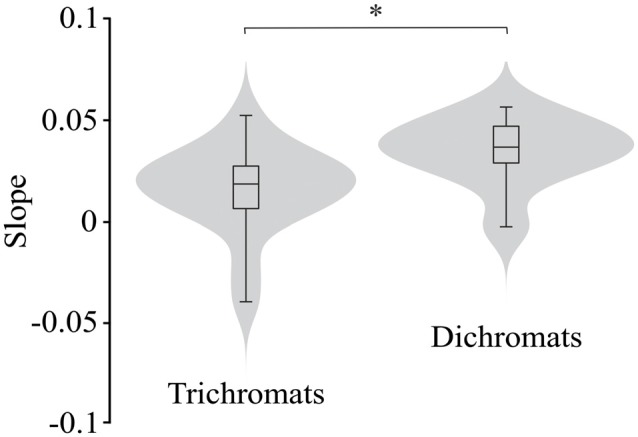
Violin plots comparing the slopes estimated from the best individual linear fits to the ellipse area as a function of the luminance noise amplitude from trichromats and congenital dichromats. Dichromats had larger slopes than trichromats. Shaded gray areas are the kernel density plot that represents 95% of the data distribution, and the box plot comprises horizontal lines that represent third quartile (upper line), median (middle line), first quartile (lower line) and whiskers represents the maximum and minimum values. *p < 0.05.

**Figure 8:**
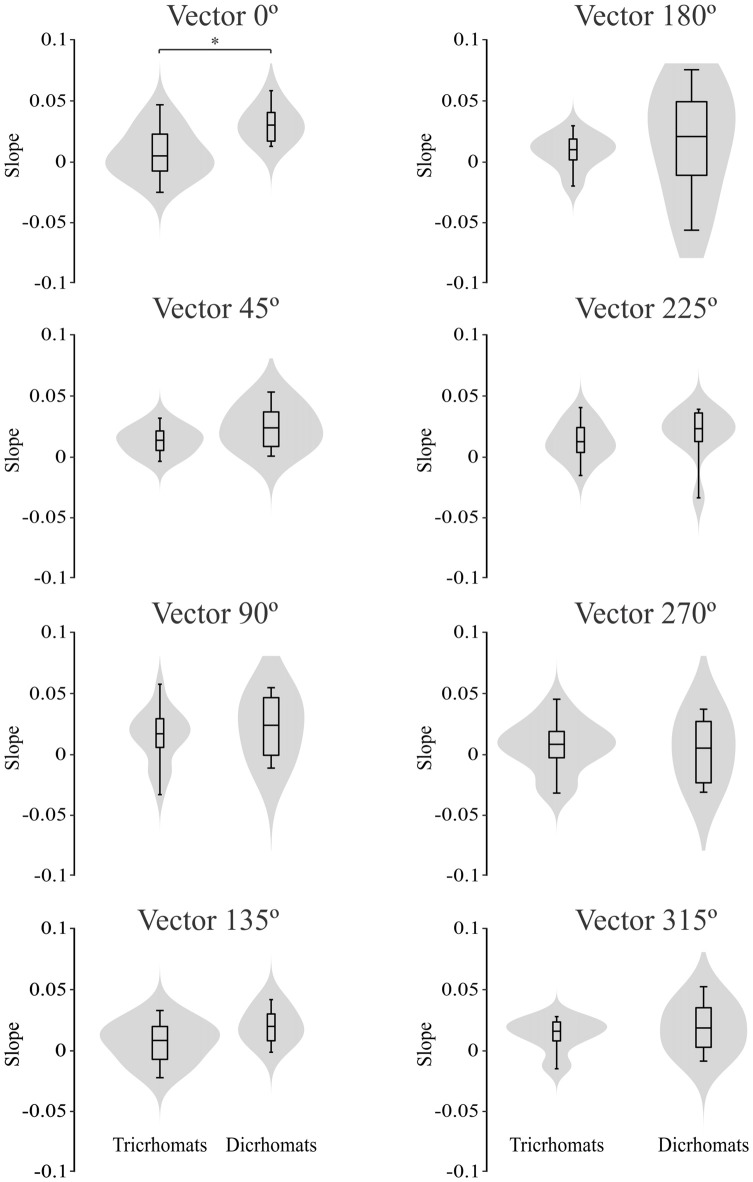
Violin plots comparing the slopes estimated from the best individual linear fits to the chromatic vector thresholds as a function of the luminance noise amplitude from trichromats and congenital dichromats. Only at chromatic vector 0°, the slopes estimated from functions obtained of dichromats were larger than those obtained from trichromats. Shaded gray areas are the kernel density plot that represents 95% of the data distribution, and the box plot comprises horizontal lines that represent third quartile (upper line), median (middle line), first quartile (lower line) and whiskers represents the maximum and minimum values. *p < 0.05.

Figure 7 illustrates the violin plots comparing the slope of the fitted lines to the ellipse area as a function of luminance noise amplitude for the trichromats and dichromats. It was observed that the functions from dichromats data had larger slopes than those from trichromats (Mann-Whitney test, *p* < 0.05). For the chromatic vectors, only in the chromatic axes 0°, a statistically significant difference was observed between the slopes of the fitted lines to the chromatic discrimination data as a function of the luminance noise amplitude (Figure 8, *H*_(15)_ = 27.31, *p* = 0.03). These data showed that the dichromats had larger slopes than the trichromats (*p* = 0.003). Table 1 shows the linear regression results between chromatic thresholds and luminance noise amplitude.

## Discussion

In this current study the main result was luminance noise changes of a pseudoisochromatic stimulus had a greater influence on color discrimination in congenital dichromats than in trichromats. Moreover, there was a preferential effect at the chromatic axes between the deutan and protan color confusion lines.

Changes in the luminance noise have been shown to have significant influence on color discrimination in normal and anomalous trichromats (Souza et al., 2014; Cormenzana Méndez et al., 2016; Linhares et al., 2016). Previous data reported from our laboratory, compared stimuli with different quantities of luminance noise levels and observed that color discrimination of normal trichromats improved with a greater number of levels (Souza et al., 2014). We also observed that color discrimination followed Weber’s contrast between the maximum and minimum noise luminances when the mean luminance of the stimulus was changed (Cormenzana Méndez et al., 2016). Linhares et al. (2016) observed that dynamic luminance noise improved color discrimination of anomalous trichromats and had no significant effect in the color discrimination of normal trichromats. In the present investigation, as the luminance noise range decreased, Weber’s contrast of the noise and value of local luminance contrasts also decreased, while the color discrimination improved. It is not clear which indicator (Weber’s contrast, local luminance contrast or even some homogeneity measurements of the luminance noise) or combination of indicators is more important to influence the color discrimination using a pseudoisochromatic stimuli.

Luminance noise in the chromatic stimulus was introduced by Stilling (1877) to exclude or decrease a bias on color discrimination due to different brightness from different colors (Stilling, 1877; Mollon, 2003). In our experimental set up, the decrease of luminance noise range made luminance noise more homogeneous and thus probably facilitated discriminating target from the mosaic field. For trichromats, we observed a small decay in the ellipse area as the luminance range decreased, although this change was non-significant. Also in this current study, a significant difference was observed between the conditions with higher and lower luminance range in congenital dichromats, and there was a significant difference between the slopes estimated from congenital dichromats and trichromats. It is reasonable to assume that the decrease in luminance noise range has facilitated color discrimination in congenital dichromats.

In a stimulus with high homogeneity in luminance values (low luminance noise range), the difference between the target and the mosaic field could be defined both in chromaticity (absent for congenital dichromats, but present for normal trichromats) and in apparent color brightness (present for congenital dichromats and trichromats). We interpreted the increased performance in the low luminance range condition as mainly due to the Helmholtz-Kohlrausch effect, in which two colors with the same luminance but different chroma have different apparent brightness (Wyszecki and Stiles, 1967; Nayatani, 1998; Corney et al., 2009). Supposedly, the performances of both trichromats and dichromats benefited from the Helmholtz-Kohlrausch effect (a perceptual bias that motivates the use of luminance noise in the first place), but the facilitation was more evident for dichromats since trichromats already displayed high discrimination at all chromaticity vectors.

An additional result, that confirmed our interpretation that luminance bias contributes to target discrimination at low luminance range conditions, was that we could only observe the improvement of the color discrimination performance in the congenital dichromats for the half of the chromatic vectors. The other vectors also had a better performance, however they were not statistically significant. In the conditions of low luminance noise range, some apparent color brightness differences could be present and possibly assisted them to determine the correct targets for the chromatic axes from 0° to 135°. For the other chromatic vectors, as well as for all chromatic vectors in trichromats, the difference in chromaticity alone was enough to guide discrimination at all luminance noise range conditions.

Our new findings reaffirmed our previous suggestions that a better description of the pseudoisochromatic stimulus parameters is necessary to make results of color discrimination tasks from different scientific laboratories more comparable.

## Author Contributions

All authors contributed to the conception of the work, and in drafting/revising the manuscript. All authors have approved the final version and agree to be accountable for all aspects of the work. GSS designed the experiments with help of TdL and LM. The psychophysical experiments were carried out by TdL, KB, GS, FB and RS. The molecular biology was carried out by DB, LB and EH and the data were analyzed by LM and TdL. Components of the manuscript were written by GSS, TdL, PG, MC, DV and MF. PG, MC, DV and MF commented on the manuscript and aided in the interpretation of the data.

## Conflict of Interest Statement

The authors declare that the research was conducted in the absence of any commercial or financial relationships that could be construed as a potential conflict of interest. The reviewer TC declared a past co-authorship with one of the authors DV to the handling editor.

## References

[B1] Corney D. Haynes J. D. Rees G. Lotto R. B. (2009). The brightness of colour. PLoS One 4:e5091. 10.1371/journal.pone.0005091 19333398 PMC2659800

[B2] Cormenzana Méndez I. Martín A. Charmichael T. L. Jacob M. M. Lacerda E. M. Gomes B. D.. (2016). Color discrimination is affected by modulation of luminance noise in pseudoisochromatic stimuli. Front. Psychol. 7:1006. 10.3389/fpsyg.2016.01006 27458404 PMC4934133

[B3] Khachiyan L. G. (1979). A polynomial algorithm in linear programming. Dokl. Akad. Nauk SSSR 244, 1093–1096.

[B4] Linhares J. M. M. João C. A. R. Silva E. D. G. de Almeida V. M. N. Santos L. A. Álvaro L.. (2016). Assessing the effects of dynamic luminance contrast noise masking on a color discrimination task. J. Opt. Soc. Am. A 33, 178–183. 10.1364/JOSAA.33.00A178 26974922

[B5] Mollon J. D. (2003). “The origins of modern color science,” in Color Science, ed. Shevell S. (Washington, DC: Optical Society of America), 1–39.

[B6] Moura A. L. Teixeira R. A. Oiwa N. N. Costa M. F. Feitosa-Santana C. Callegaro D.. (2008). Chromatic discrimination losses in multiple sclerosis patients with and without optic neuritis using the Cambridge Colour Test. Vis. Neurosci. 25, 463–468. 10.1017/s0952523808080437 18598419

[B7] Nayatani Y. (1998). A colorimetric explanation of the helmholtz-kohlrausch effect. Col. Res. Appl. 23, 374–378. 10.1002/(SICI)1520-6378(199812)23:6<374::AID-COL5>3.0.CO;2-W

[B8] Neitz M. Neitz J. (1995). Numbers and ratios of visual pigment genes for normal red-green color vision. Science 267, 1013–1016. 10.1126/science.7863325 7863325

[B9] Paramei G. V. (2012). Color discrimination across four life decades assessed by the Cambridge Colour Test. J. Opt. Soc. Am. A 29, A290–A297. 10.1364/JOSAA.29.00A290 22330391

[B10] Paramei G. V. Oakley B. (2014). Variation of color discrimination across the life span. J. Opt. Soc. Am. A 31, A375–A384. 10.1364/JOSAA.31.00A375 24695196

[B11] Regan B. C. Reffin J. P. Mollon J. D. (1994). Luminance noise and the rapid determination of discrimination ellipses in colour deficiency. Vis. Res. 34, 1279–1299. 10.1016/0042-6989(94)90203-8 8023437

[B12] Shinomori K. Panorgias A. Werner J. S. (2016). Discrimination thresholds of normal and anomalous trichromats: model of senescent changes in ocular media density on the Cambridge Colour Test. J. Opt. Soc. Am. A 33, A65–A76. 10.1364/JOSAA.33.000A65 26974943 PMC5316232

[B13] Souza G. S. Malone F. L. Crawford T. L. Miquilini L. Salomão R. C. Guimarães D. L.. (2014). Low number of luminance levels in the luminance noise increases color discrimination thresholds estimated with pseudoisochromatic stimuli. Front. Psychol. 5:1291. 10.3389/fpsyg.2014.01291 25566106 PMC4274881

[B14] Stilling J. (1877). Die Prüfung des Farbensinnes beim Eisenbahn- und Marine-Personal. Cassel: Theodor Fischer.

[B15] Wyszecki G. Stiles W. S. (1967). Color science: concepts and methods, quantitative data and formulas. J. SMPTE 76:1154 10.5594/J13680

